# Glycan Microarrays as Chemical Tools for Identifying Glycan Recognition by Immune Proteins

**DOI:** 10.3389/fchem.2019.00833

**Published:** 2019-12-13

**Authors:** Chao Gao, Mohui Wei, Tanya R. McKitrick, Alyssa M. McQuillan, Jamie Heimburg-Molinaro, Richard D. Cummings

**Affiliations:** Department of Surgery, National Center for Functional Glycomics, Beth Israel Deaconess Medical Center, Harvard Medical School, Boston, MA, United States

**Keywords:** glycans, microarrays, glycoimmunology, glycan-binding proteins, immunology, immune receptors, immune proteins

## Abstract

Glycans and glycan binding proteins (GBPs or lectins) are essential components in almost every aspect of immunology. Investigations of the interactions between glycans and GBPs have greatly advanced our understanding of the molecular basis of these fundamental immunological processes. In order to better study the glycan-GBP interactions, microscope glass slide-based glycan microarrays were conceived and proved to be an incredibly useful and successful tool. A variety of methods have been developed to better present the glycans so that they mimic natural presentations. Breakthroughs in chemical biology approaches have also made available glycans with sophisticated structures that were considered practically impossible just a few decade ago. Glycan microarrays provide a wealth of valuable information in immunological studies. They allow for discovery of detailed glycan binding preferences or novel binding epitopes of known endogenous immune receptors, which can potentially lead to the discovery of natural ligands that carry the glycans. Glycan microarrays also serve as a platform to discover new GBPs that are vital to the process of infection and invasion by microorganisms. This review summarizes the construction strategies and the immunological applications of glycan microarrays, particularly focused on those with the most comprehensive sets of glycan structures. We also review new methods and technologies that have evolved. We believe that glycan microarrays will continue to benefit the growing research community with various interests in the field of immunology.

## Introduction

Immunology as a field is continuously evolving and expanding. With thousands of publications every year, it fundamentally impacts many aspects of biomedical sciences and has led to fascinating breakthroughs in a large number of biomedical areas, including primary immunodeficiencies, immunometabolism, neuroimmunology, mucosal immunology, cancer immunotherapy, and vaccine development. It is becoming increasingly clear that major components of our immune system include glycoproteins and recognition of glycans by glycan-binding proteins (GBPs) or lectins (Rabinovich et al., 2012; Colomb et al., 2019; Läubli and Varki, 2019; Pascoal et al., 2019; Taylor and Drickamer, 2019). These components are involved in all aspects of cellular recognition and signaling, both biological and pathological. Glycans are essential modulators in both innate and adaptive immune systems. They are binding ligands for innate immune receptors, such as selectins, galectins, and Siglecs, many of which are targets for cancer immunotherapy (RodrÍguez et al., 2018). Glycans are also indirectly involved in the protein-protein interactions by contributing to protein conformations and oligomerization. Immune checkpoint molecules PD-1 (Okada et al., 2017) and PD-L1 (Li et al., 2018; Lee et al., 2019) are both heavily glycosylated, and the glycans are required for their normal interactions and subsequent suppression of T cell activities. In addition, human glycans are receptors of surface proteins of pathogens, such as bacteria, fungi and viruses that are involved in virtually all types of infectious disease processes (Li et al., 2017; Byrd-Leotis et al., 2019b).

Identifying the interactions between glycans and GBPs is, therefore, key to understanding the molecular mechanisms of these immunological events. There is a need for facile methods to study such interactions in a miniaturized and high throughput manner. A major breakthrough in this effort was the development of printed glycan microarrays (Fukui et al., 2002; Wang et al., 2002; Blixt et al., 2004; Geissner et al., 2019), which enable simultaneous binding analyses of GBPs to hundreds of glycan structures. The public availability of the glycan microarrays built by the Consortium for Functional Glycomics (www.functionalglycomics.org, CFG) facilitated the analyses of many GBPs in various aspects of immunology. With the technology developments at the National Center for Functional Glycomics (NCFG), these key immunological players have been re-examined with a larger variety of glycan sequences. This review introduces glycan microarray technologies from a historical point of view, summarizes the recent developments in the chemical and chemoenzymatic methods and new glycan microarrays with the focus on the CFG and NCFG approaches. We highlight the biological applications of glycan microarrays in immunology studies, and discuss current challenges and future perspectives of this technology.

## Historical Aspects of Binding Assays to Probe Glycan-GBP Interactions

Early efforts in the investigation of glycan-GBP interactions involved indirect methods such as hemagglutination inhibition and inhibition of precipitation assays. In these assays, free monosaccharides or oligosaccharides were tested as inhibitors against the binding between glycan-bearing substances, such as red blood cells or glycoprotein-containing extracts, and GBPs such as plant lectins and antisera. These methods led to the discovery of major blood group A, B, O (H) epitopes by Morgan and Watkins, and Kabat and colleagues between the 1950's and 1970's (Watkins, 2001). They have been adapted to different fields of biological research and are still widely used nowadays. For instance, hemagglutination inhibition assay is routinely used to determine the titer of antibodies against influenza viruses in human serum.

Solid phase direct binding assays were developed thereafter, in which GBPs were directly radio-labeled and the binding assays were performed on thin-layer chromatography (TLC). Breakthrough discoveries in this stage include the identification of GM1 as the ligand for cholera toxin (Magnani et al., 1980), the pancreatic cancer-associated biomarker CA19-9 (Magnani et al., 1982) and stage-specific embryonic antigens (Gooi et al., 1981; Kannagi et al., 1982, 1983). This method was superseded by EL‘-based binding assays in which secondary proteins were enzyme-conjugated or fluorescently labeled. Glycans or glycopeptides labeled with biotin can be immobilized to wells of a microtiter plate pre-coated with streptavidin and directly assayed (Blixt et al., 2003; Alvarez and Blixt, 2006). This assay format enabled the comparison of the binding activities of dozens of glycans at the same time and is the prototype of glycan microarrays. The results revealed many features on the binding specificities of P- and L-selectin with PSGL-1 glycopeptide (Leppänen et al., 2002, 2003), and of galectin-1 (Leppänen et al., 2005).

One drawback of prior methods is that they required relatively large quantities of GBPs and often milligram levels of glycans. Due to their structural heterogeneity and complexity, the glycans cannot be easily acquired either by isolation and purification from natural sources, or by synthetic routes using chemical synthesis or chemoenzymatic synthesis. Moreover, the diversity of the glycan structures with sufficient amounts available for study is not comparable to the diversity of glycans in nature. It is estimated that the human glycome contains ~3,000 glycan species or more on glycoproteins and glycolipids, and ~4,000 theoretical pentasaccharide sequences on GAGs (Cummings, 2009) but only a small proportion of these sequences are currently available for bioactivity studies. Both of these factors highlight the demand for a microarray platform with structurally diverse glycans and minimal sample consumption, thus marks the era of glycan microarray.

Since their invention, glycan microarrays have become an essential tool in glycobiology, with increasing number and diversity of glycans and extremely small amounts of glycan consumption (Li and Feizi, 2018; Cummings, 2019). In particular, the glycan microarrays provided by NCFG and CFG not only revealed the fine binding specificities of known GBPs, they also helped in identification of novel GBPs and their novel biological activities through collaborative projects with investigators all over the world, which, altogether led to hundreds of published papers. The arrays have also served as a screening tool to provide investigators with leads to follow in subsequent assays that delve deeper into binding interactions between glycans and their immune protein of interest.

## Chemistry Aspects of Glycan Microarrays

The key step to creating glycan microarrays is to establish proper methods to immobilize the glycans onto the solid phase, via either non-covalent or covalent approaches. There have been extensive reviews discussing the available chemical methods for glycan immobilization (Fukui et al., 2002; Rillahan and Paulson, 2011; Park et al., 2013; Palma et al., 2014; Song et al., 2014, 2015).

The strategy taken by the CFG and NCFG to generate glycan microarrays is covalent attachment, which requires glycans to be covalently derivatized with a specific bi-functional chemical group that can subsequently be used to covalently react with a functionalized solid surface. Although glycans produced by *de novo* chemical or chemo-enzymatic synthesis are usually provided with a reactive handle to allow subsequent immobilization, free reducing glycans, such as milk oligosaccharides or natural glycans released enzymatically, need proper derivatization to enable quantification and attachment. The ability to label glycans from natural sources is of particular importance, as it allows the field to overcome the limitations of synthetic routes and vastly expand the repertoire of glycans that could be incorporated into the microarray platform. Due to the wide availability of N-hydroxysuccinimide (NHS) ester- or epoxy-derivatized glass slides, much effort has been devoted to the development of amine-containing bi-functional linkers, which can be coupled to the reducing end of the glycan and contains a reactive amine group to covalently attach to solid supports.

### Development of Fluorescent Bi-Functional Linker

**Figure 1:**
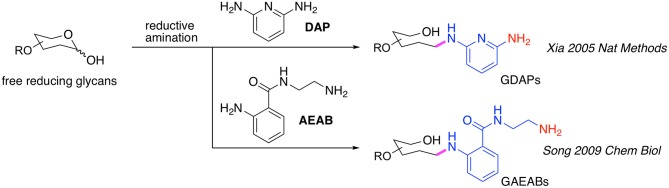
Bifunctional fluorescent glycan linkers developed in the Cummings Lab.

Initially, the commercially available 2,6-diaminopyridine (DAP) (Xia et al., 2005) was used to generate glycan-DAP conjugates (GDAPs) that are fluorescent and contain a primary aryl amine for immobilization (Figure 1). A wide variety of glycans were successfully converted to GDAPs which were reactive to NHS-activated surface, maleimide-activated protein, carboxylated microspheres and NHS-biotin, demonstrating the general utility of DAP for glycan labeling and glycan microarray construction. However, these DAP derivatives showed higher immobilization efficiency on epoxy slides compared to NHS-activated glass (Song et al., 2008). The low reactivity of the aromatic amine of DAP and the weak fluorescence restricted the utility of GDAPs.

Song et al. therefore developed another bifunctional fluorescent linker, 2-amino-N-(2-amino-ethyl)-benzamide (AEAB, Figure 1) (Song et al., 2009). AEAB selectively reacts with free reducing glycans through its aryl amine to form glycan-AEAB conjugates (GAEABs). The remaining primary alkyl amine is suitable for efficient immobilization on epoxy or NHS slides, and therefore makes it a great linker for glycan microarray construction. Moreover, due to its high fluorescence sensitivity and conjugation yield, AEAB is ideal for development of natural glycan microarrays or shotgun glycomics, whereby all types of glycans could be isolated from natural sources and be fluorescently-tagged, purified by multi-dimensional chromatography, quantified and eventually printed on glass slides to create natural glycan microarrays. With this approach, we developed a variety of sequence-defined and shotgun glycan microarrays, including a human milk glycan array, a microbial glycan microarray (MGM), sequence-defined and shotgun Schistosome glycan arrays, a pig lung N-glycan array and more recently, a sequence-defined NCFG array, a lectin QA/QC array and a human lung shotgun N-glycan array (Byrd-Leotis et al., 2019b).

It should be noted that another approach using 2-aminobenzamide (2-AB) and 2-aminobenzoic acid (2-AA) has also been developed, whereby glycans reductively labeled with 2-AB can be isolated by chromatography and then directly covalently coupled to epoxy-activated glass slides via secondary amine chemistry to generate 2-AB or 2-AA-glycan microarrays (de Boer et al., 2007; van Diepen et al., 2015). These approaches may be considered equivalent to the use of AEAB and DAP.

A major drawback of using DAP, AEAB, 2-AA, and 2-AB is that their conjugation reaction relies on reductive amination which, although highly efficient, opens the sugar ring at the reducing end monosaccharide (Figure 1). This destroys the reducing end integrity of the glycan (Prasanphanich et al., 2015) and potentially results in non-natural presentation of glycans on the array. This would affect the binding affinities of smaller glycans, and particularly those with modifications in the core region, such as core Fuc on N-glycans (Prasanphanich et al., 2015). We, therefore, have been developing new strategies to overcome this weakness while retain the merits mentioned above.

### New Strategies to Display Sequence-Defined and Natural Glycans

#### F-MAPA

**Figure 2:**
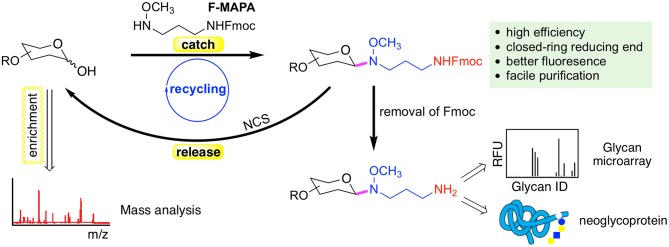
A reversible fluorescent linker F-MAPA recently developed in the Cummings Lab. The linker enables highly efficient conjugation of glycans with their reducing end preserved. The Fmoc group in the F-MAPA linker renders strong fluorescence and hydrophobicity, both of which facilitates the purification. The Fmoc (fluorenylmethyloxycarbonyl) can be easily removed to support further manipulation and applications such as glycan microarrays and neoglycoprotein synthesis. The F-MAPA can also be cleaved upon treatment of *N*-chlorosuccinimide (NCS) to regenerate free reducing glycans. This figure is reprinted (adapted) with permission from Wei et al. (2019) Copyright 2019 American Chemical Society.

N-alkyl oxime can react with hemiacetal of reducing glycans and form intact closed-ring reducing end, thereby preserving the integrity of glycans. We have developed a reversible fluorescent linker F-MAPA, which contains an N-alkyl oxime active motif and an Fmoc protected alkyl amine (Wei et al., 2019). F-MAPA can efficiently derivatize reducing glycans via N-alkyl oxime mediated ligation and preserve the structure integrity (Figure 2). Fmoc serves as a transient fluorophore for monitoring and quantification, as well as a hydrophobic tag for glycan enrichment, separation and purification via C-18 solid phase extraction (SPE). Fmoc also can be easily removed to generate an active alkyl amine, enabling further manipulation and application of glycans for multiple purposes (e.g., glycan microarray, neoglycoprotein synthesis, etc.).

F-MAPA is more versatile than ordinary bi-functional linkers. It can be facilely cleaved under mild conditions, such as treatment by *N*-chlorosuccinimide (NCS) to regenerate free reducing glycans, which particularly facilitates glycomics and functional glycomics. This “catch and release” approach provided by F-MAPA enables selective enrichment of free reducing glycans from complex biological samples, which can be followed by mass spectrometry (MS)-based structural analysis upon release of the linker, and parallel microarray binding analysis upon release of Fmoc. Moreover, the successful recycling of the reaction system and synthesis of neoglycoprotein indicate that F-MAPA has great potential for industrial application in the synthesis of neoglycoprotein therapeutics and vaccines. The facile and scalable synthesis, high conjugation efficiency and operational simplicity of the derivatization reaction make the linker highly accessible to general research laboratories, thereby enabling general accessibility of glycan microarrays to the research community.

#### Fmoc-Labeled Asparagine

Because of the key consideration to preserve as much as possible the natural linkage of a glycan to its aglycone, e.g., amino acid, another approach we recently developed was the chemoenzymatic synthesis using a naturally occurring amino acid as the linker (Gao et al., 2019). Due to the structural complexity and heterogeneity, *de novo* chemical synthesis or isolation from natural sources had been intimidating for N-glycan production. Although chemoenzymatic synthesis has provided an alternative route, many of the methods rely on sophisticated chemical synthesis to produce the core structures to start with. These suffered from low yield and efficiency due to the usage of non-mammalian glycosyltransferases and poor substrates in which the aglycone potentially interferes with the reactivity. Moreover, evidence has shown that the linkers also play a role in microarray binding experiments (Padler-Karavani et al., 2012; Tessier et al., 2013; Grant et al., 2014).

As the natural N-glycan carrier, asparagine (Asn) guarantees the beta-configuration of the N-glycans, thus at least partially retains the natural presentation of N-glycans within context of N-glycopeptides. Asn-linked biantennary N-glycan core is abundant in chicken egg yolk. Fmoc can be selectively installed on the primary amine of the asparagine residue. Similar to F-MAPA, it renders strong fluorescence, adequate hydrophobicity and can be easily removed. The Asn-linked N-glycans have also been shown to be great substrates for enzymatic reactions. They are often quantitatively converted to products with the presence of mammalian glycosyltransferases.

Using this approach, we successfully synthesized a library of 32 multiantennary Asn-linked N-glycans and prepared the Asn-linked N-glycan array (designated N-glycan array). All of them are naturally occurring complex N-glycan structures found in human and other mammals. We will discuss the application of this glycan microarray in detail in the next section.

### Future Directions in Linker Development

The Asn-linked chemoenzymatic approach holds great promise in expansion to more asymmetric complex N-glycan structures. In combination with specifically designed sugar donors, it is now possible to generate asymmetric multiantennary N-glycans without specially synthesized core structures in the initial steps, and thus can significantly reduce the difficulty in total chemical synthesis of N-glycans (Liu et al., 2019). We envisage that there will soon be N-glycan arrays with extremely complex structures available for function assays.

Versatile as it is, the F-MAPA does not contain a fluorophore on the backbone but rather, relies on the Fmoc protecting group attached to the primary amine. Thus, an extra step to remove Fmoc is needed prior to any further treatments. This also applies to the natural amino acid linker Asn. Therefore, it would be ideal to develop new linkers with fluorophores that do not require these additional steps.

One obstacle in studying glycan-GBP interaction is the potentially low affinity of some GBPs for certain types of glycans. In the natural state, the glycan-GBP interaction often occurs in a multivalent fashion, accomplished by clustered presentation of ligands and/or carbohydrate recognition domains. Many labs have, thus, developed natural protein or synthetic polymers to generate glycan microarrays (Maierhofer et al., 2007; Jayaraman, 2009; Huang et al., 2015; Mende et al., 2019). It would be beneficial to have easily accessible and facile technologies to generate linker-bearing scaffolds to achieve higher binding avidity in the future.

## Application of Glycan Microarrays in Immunology

With the above-mentioned chemical methods in position, we have generated glycan microarrays that encompass various types of glycan structures to facilitate the biological studies both from our group at the NCFG and through the use of the CFG glycan microarray by investigators world-wide. We summarize below a few with highlights on their applications in addressing key aspects of immunological studies.

### Natural Ligands for Endogenous Immune Receptors

The innate immune system recognizes pathogens and self-antigens by endogenous receptors expressed on the surface of or secreted by immune cells. A large variety of these receptors have been identified, including Siglecs, galectins and C-type lectins. Typically, they recognize the pathogen-associated and damage-associated molecular patterns (PAMPs and DAMPs, respectively), as well as self-associated molecular patterns (SAMPs) and many of these interactions involve binding to carbohydrates. Due to their myriad of functions in anti-microbial defense and immune homeostasis, understanding of the fine binding specificities of these receptors is critical in our understanding of how the innate immune system responds selectively in the context of infections, autoimmunity and cancer.

#### Siglecs

Siglecs are a family of sialic acid-binding lectins that are mostly located on the surface of hematopoietic cells. They are involved in cell signaling and adhesion, and are particularly important in immune cell regulation (Bornhofft et al., 2018). There are 14 different Siglecs in the human genome. The detailed binding specificity of these Siglecs was mainly investigated in 2000's (Blixt et al., 2003; Campanero-Rhodes et al., 2006; Crocker et al., 2007) and early 2010's (Macauley et al., 2014) when only a limited number and diversity of glycans were available. Particularly, their binding preferences to sialic acid linkage in the context of N-glycans were not fully characterized due to a lack of paired multiantennary complex-type N-glycans. Therefore, we sought to take a systematic chemoenzymatic approach for N-glycan synthesis and address the specificities by microarray analyses.

**Figure 3:**
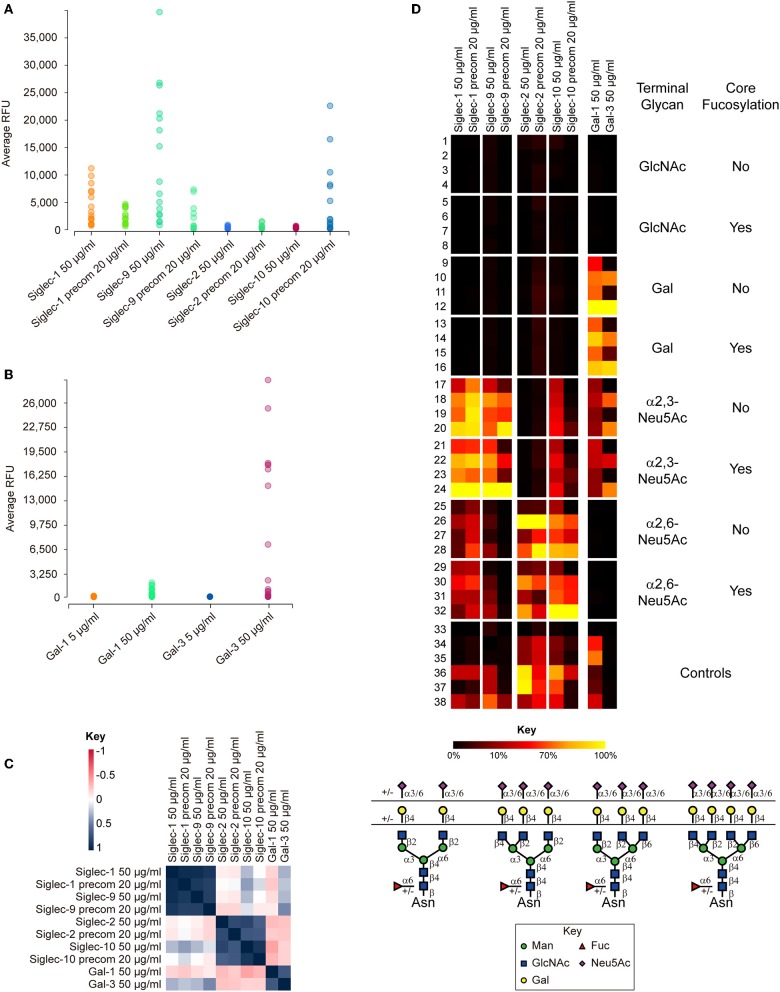
Summary of the binding results of human Siglecs-1, -2, -9, -10, galectin-1 (Gal-1), and galectin-3 (Gal-3) on N-glycan microarray. **(A)** Binding RFUs of Siglecs tested at 50 μg/ml without precomplex with the secondary antibody, or at 20 μg/ml with precomplex to sixteen sialylated N-glycans on the microarray. **(B)** Binding RFUs of galectins tested at 5 or 50 μg/ml to all complex N-glycans on the array. Each colored bubble represents the binding signal elicited by one N-glycan. **(C)** Correlation of the overall binding results between samples on the N-glycan microarray. The Siglec-1 and 9, Siglec-2 and 10, Galectin-1 and -3 are segregated into three different groups, suggesting similar binding patterns found within each group. The key represents the correlation coefficiency between two samples. **(D)** Relative binding intensities of the Siglecs and galectins. The RFUs of the top binder of each protein was set 100% (colored in yellow). Color reflects the relative binding intensity relative to the strongest signal within the results of each protein. The N-glycans were grouped according to the structural features listed on right: GlcNAc, Gal, α2,3-Neu5Ac, and α2,6-Neu5Ac, without or with core Fuc. Within each subgroup, the glycans were listed according to the branching patterns in the order of biantennary, 224-triantennary, 226-triantennary and tetraantennary as shown at the bottom. The data was processed and presented by the GLAD toolkit (https://www.glycotoolkit.com/Tools/GLAD/) developed in house (Mehta and Cummings, 2019). precom, precomplex; RFU, relative fluorescent units; GLAD, GLycan Array Dashboard.

We synthesized an array of Asn-linked isomeric multiantennary N-glycans with varying terminal non-reducing sialic acid, galactose, and N-acetylglucosamine residues, as well as core fucose (Figure 3). Using this microarray, and together with the sequence-defined glycan array from CFG, we investigated 11 human Siglecs, Siglec-1 to Siglec-11, and a rat-derived Siglec-4, all in human Fc chimera form, with particular focus on their binding to multiantennary N-glycans. As the affinity of Siglec-ligand interaction is generally thought to be low, we also performed the binding assays with Siglecs precomplexed with the secondary antibodies to increase the binding avidity.

Of the twelve Siglecs tested, only four showed binding to the N-glycan array (Figure 3A). Siglec-1 and-9 preferentially recognized glycans containing the determinant NeuAcα2-3Galβ1-4GlcNAc-, irrespective of the presence of core fucosylation (Figure 3D). The binding was independent of the branching patterns, but there was a clear trend that glycans with more α2,3-linked NeuAc elicited stronger signals. The binding of the two Siglecs without precomplex was comparable to or higher than with precomplex, suggesting that the affinities of Siglec-1 and-9 are high (Figure 3A). In contrast to Siglec-1 and-9, we observed that Siglec-2 and-10 both preferentially bound N-glycans terminating in α2,6-linked sialic acid, although some weak binding of Siglec-10 was detected to N-glycans with α2,3-linked sialic acid (Figures 3C,D). Preincubation with the secondary antibody significantly increased the binding intensity of Siglec-10, suggesting multivalency may be important to this protein (Figure 3A). This was also reflected by the observation that glycans containing higher numbers of branches (and thus more binding epitopes) elicited higher binding signals. Interestingly, no binding was detected with the other Siglecs on the N-glycan array, indicating their lack of recognition of N-glycans in these formats.

Collectively, our data indicates that sialylated, multiantennary N-glycans are potential endogenous receptors for Siglecs-1,-2,-9, and -10. These results shed lights on the biological functions of these Siglecs considering the potential high density of these natural N-glycan ligands on the cell surface.

In order to compare our results to those previously published, we tested the Siglecs on the CFG glycan microarray, one of the most comprehensive glycan microarray screening platforms. This array provides a great tool for researchers to determine if their protein of interest has glycan binding capabilities. The most current version of the CFG slides contains 585 unique, diverse mammalian-type glycans that are either synthetically made or chemoenzymatically modified. The glycans have amino linkers, of which there are twenty distinct structures, which are covalently attached to NHS-activated glass microscope slides. This array consists of a number of different classes of glycans that are common epitopes observed in biological interactions, among which there are 114 N-linked glycans with 28 sialylated species (summarized in Table 1).

**Table 1:** All of the sialylated N-glycans on the CFG array; α2,3- and α2,6-linked sialic acids are colored in blue and red, respectively.

**ID**	**Glycan sequence**
341	Neu5Acα2-6Galβ1-4GlcNAcβ1-2Manα1-6Manβ1-4GlcNAcβ1-4GlcNAcβ-Sp12
342	Neu5Acα2-6Galβ1-4GlcNAcβ1-2Manα1-3Manβ1-4GlcNAcβ1-4GlcNAcβ-Sp12
339	Neu5Acα2-6Galβ1-4GlcNAcβ1-2Manα1-6(Manα1-3)Manβ1-4GlcNAcβ1-4GlcNAcβ-Sp12
340	Manα1-6(Neu5Acα2-6Galβ1-4GlcNAcβ1-2Manα1-3)Manβ1-4GlcNAcβ1-4GlcNAcβ-Sp12
304	Neu5Acα2-6Galβ1-4GlcNAcβ1-2Manα1-6(GlcNAcβ1-2Manα1-3)Manβ1-4GlcNAcβ1-4GlcNAcβ-Sp12
297	Neu5Acα2-6Galβ1-4GlcNAcβ1-2Manα1-6(Galβ1-4GlcNAcβ1-2Manα1-3)Manβ1-4GlcNAcβ1-4GlcNAcβ-Sp12
315	Galβ1-4GlcNAcβ1-2Manα1-6(Neu5Acα2-6Galβ1-4GlcNAcβ1-2Manα1-3)Manβ1-4GlcNAcβ1-4GlcNAcβ-Sp12
55	Neu5Acα2-6Galβ1-4GlcNAcβ1-2Manα1-6(Neu5Acα2-6Galβ1-4GlcNAcβ1-2Manα1-3)Manβ1-4GlcNAcβ1-4GlcNAcβ-Sp12
56	Neu5Acα2-6Galβ1-4GlcNAcβ1-2Manα1-6(Neu5Acα2-6Galβ1-4GlcNAcβ1-2Manα1-3)Manβ1-4GlcNAcβ1-4GlcNAcβ-Sp21
57	Neu5Acα2-6Galβ1-4GlcNAcβ1-2Manα1-6(Neu5Acα2-6Galβ1-4GlcNAcβ1-2Manα1-3)Manβ1-4GlcNAcβ1-4GlcNAcβ-Sp24
314	Neu5Acα2-6Galβ1-4GlcNAcβ1-2Manα1-6(Neu5Acα2-3Galβ1-4GlcNAcβ1-2Manα1-3)Manβ1-4GlcNAcβ1-4GlcNAcβ-Sp12
320	Neu5Acα2-3Galβ1-4GlcNAcβ1-2Manα1-6(Neu5Acα2-3Galβ1-4GlcNAcβ1-2Manα1-3)Manβ1-4GlcNAcβ1-4GlcNAcβ-Sp12
321	Neu5Acα2-3Galβ1-4GlcNAcβ1-2Manα1-6(Neu5Acα2-6Galβ1-4GlcNAcβ1-2Manα1-3)Manβ1-4GlcNAcβ1-4GlcNAcβ-Sp12
474	Neu5Acα2-6Galβ1-4GlcNAcβ1-2Manα1-6(Neu5Acα2-6Galβ1-4GlcNAcβ1-2Manα1-3)Manβ1-4GlcNAcβ1-4(Fucα1-6)GlcNAcβ-Sp24
475	Neu5Acα2-3Galβ1-4GlcNAcβ1-2Manα1-6(Neu5Acα2-3Galβ1-4GlcNAcβ1-2Manα1-3)Manβ1-4GlcNAcβ1-4(Fucα1-6)GlcNAcβ-Sp24
452	Neu5Acα2-3Galβ1-4GlcNAcβ1-2Manα1-6(GlcNAcβ1-4)(Neu5Acα2-3Galβ1-4GlcNAcβ1-2Manα1-3)Manβ1-4GlcNAcβ1-4GlcNAcβ-Sp21
456	Neu5Acα2-6Galβ1-4GlcNAcβ1-2Manα1-6(GlcNAcβ1-4)(Neu5Acα2-6Galβ1-4GlcNAcβ1-2Manα1-3)Manβ1-4GlcNAcβ1-4GlcNAcβ-Sp21
478	Neu5Acα2-3Galb1-3GlcNAcβ1-2Manα1-6(GlcNAcβ1-4)(Neu5Acα2-3Galb1-3GlcNAcβ1-2Manα1-3)Manβ1-4GlcNAcβ1-4GlcNAcβ-Sp21
578	Neu5Acα2-3Galβ1-4GlcNAcb1-3Galβ1-4GlcNAcβ1-2Manα1-6(Neu5Acα2-3Galβ1-4GlcNAcb1-3Galβ1-4GlcNAcβ1-2Manα1-3)Manβ1-4GlcNAcβ1-4GlcNAcβ-Sp12
583	Neu5Acα2-6Galβ1-4GlcNAcb1-3Galβ1-4GlcNAcβ1-2Manα1-6(Neu5Acα2-6Galβ1-4GlcNAcb1-3Galβ1-4GlcNAcβ1-2Manα1-3)Manβ1-4GlcNAcβ1-4GlcNAcβ-Sp12
581	Neu5Acα2-6Galβ1-4GlcNAcb1-3Galβ1-4GlcNAcb1-3Galβ1-4GlcNAcβ1-2Manα1-6(Neu5Acα2-6Galβ1-4GlcNAcb1-3Galβ1-4GlcNAcb1-3Galβ1-4GlcNAcβ1-2Manα1-3)Manβ1-4GlcNAcβ1-4GlcNAcβ-Sp12
582	Neu5Acα2-3Galβ1-4GlcNAcb1-3Galβ1-4GlcNAcb1-3Galβ1-4GlcNAcβ1-2Manα1-6(Neu5Acα2-3Galβ1-4GlcNAcb1-3Galβ1-4GlcNAcb1-3Galβ1-4GlcNAcβ1-2Manα1-3)Manβ1-4GlcNAcβ1-4GlcNAcβ-Sp12
453	Neu5Acα2-3Galβ1-4GlcNAcβ1-4Manα1-6(GlcNAcβ1-4)(Neu5Acα2-3Galβ1-4GlcNAcβ1-4(Neu5Acα2-3Galβ1-4GlcNAcβ1-2)Manα1-3)Manβ1-4GlcNAcβ1-4GlcNAcβ-Sp21
457	Neu5Acα2-6Galβ1-4GlcNAcβ1-4Manα1-6(GlcNAcβ1-4)(Neu5Acα2-6Galβ1-4GlcNAcβ1-4(Neu5Acα2-6Galβ1-4GlcNAcβ1-2)Manα1-3)Manβ1-4GlcNAcβ1-4GlcNAcβ-Sp21
454	Neu5Acα2-3Galβ1-4GlcNAcβ1-6(Neu5Acα2-3Galβ1-4GlcNAcβ1-2)Manα1-6(GlcNAcβ1-4)(Neu5Acα2-3Galβ1-4GlcNAcβ1-2Manα1-3)Manβ1-4GlcNAcβ1-4GlcNAcβ-Sp21
458	Neu5Acα2-6Galβ1-4GlcNAcβ1-6(Neu5Acα2-6Galβ1-4GlcNAcβ1-2)Manα1-6(GlcNAcβ1-4)(Neu5Acα2-6Galβ1-4GlcNAcβ1-2Manα1-3)Manβ1-4GlcNAcβ1-4GlcNAcβ-Sp21
455	Neu5Acα2-3Galβ1-4GlcNAcβ1-6(Neu5Acα2-3Galβ1-4GlcNAcβ1-2)Manα1-6(GlcNAcβ1-4)(Neu5Acα2-3Galβ1-4GlcNAcβ1-4(Neu5Acα2-3Galβ1-4GlcNAcβ1-2)Manα1-3)Manβ1-4GlcNAcβ1-4GlcNAcβ-Sp21
459	Neu5Acα2-6Galβ1-4GlcNAcβ1-6(Neu5Acα2-6Galβ1-4GlcNAcβ1-2)Manα1-6(GlcNAcβ1-4)(Neu5Acα2-6Galβ1-4GlcNAcβ1-4(Neu5Acα2-6Galβ1-4GlcNAcβ1-2)Manα1-3)Manβ1-4GlcNAcβ1-4GlcNAcβ-Sp21

*Linkers: Sp12 = Asparagine; Sp21 = -N(CH_3_)-O-(CH_2_)_2_-NH_2_; Sp24 = KVANKT*.

**Figure 4:**
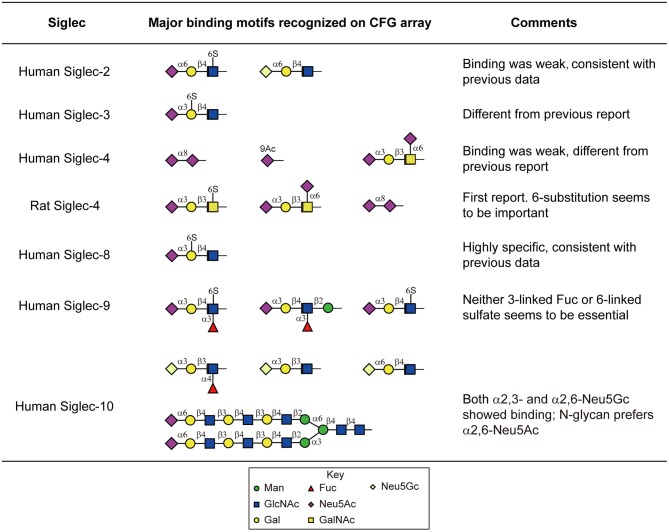
Major sialylated binding motifs bound by human and rat Siglecs on the CFG array. These results were compared to the previous report summarized by the Macauley et al. (2014) and the comments were listed in the table.

Moderate to strong binding was observed with human Siglec-3,-6,-8,-9,-10, and -11, as well as the rat Siglec-4. The others human Siglecs including-1,-2,-4,-5, and -7 showed weak or no binding. Siglecs-8 and-9 showed binding toward α2,3-sialylic acid-containing sulfated glycans (Figure 4). Siglec-8 binding was extremely strong and exclusive to Neu5Acα2-3(6S)Galβ1-4GlcNAc-. This was in accordance with the data obtained with lipid-linked glycan arrays showing that the α2,3-linked sialic acid, and the 6-sulfation on the Gal, but not on the GlcNAc, is critical (Campanero-Rhodes et al., 2006), although the exact same sequence had not been available on that array. Consistent with the previous data (Campanero-Rhodes et al., 2006; Macauley et al., 2014), the top binder of Siglec-9 was 6-sulfo-sLeX (Neu5Acα2-3Galβ1-4(Fucα1-3)(6S)GlcNAc-). This protein also exhibited strong binding to α2,3-sialic acid-containing glycans that lack either sulfate or fucose, suggesting that sulfation and fucosylation may not be essential. Similar to our data on the N-glycan array, Siglec-10 showed binding to both α2,3- and α2,6-sialylated glycans, in particular, the N-glycolyl form, Neu5Gc. However, Siglec-10 binding to N-glycans on the CFG glycan microarray, albeit weak, showed preference to the α2,6-sialylated probes (Figure 4).

Despite the weak binding intensities, Siglec-2 showed binding to the α2,6-sialic acid-containing glycans, with or without sulfation (Neu5Acα2-6Galβ1-4(6S)GlcNAc- and Neu5Gcα2-6Galβ1-4GlcNAc-, Figure 4), which suggests the possibility that Siglec-2 can indeed bind similar structures presented on N-glycans.

We also observed novel specificities that have not been reported previously. Siglec-3 bound strongly to Neu5Acα2-3(6S)Galβ1-4GlcNAc- (Figure 4), which is different from the previously reported Neu5Acα2-6Galβ1-4GlcNAc-. It has been known that the human and mouse Siglec-4 bind the α2,3-sialylated core 1 (Neu5Acα2-3Galβ1-3GalNAc-). Our data suggest that both the human and rat Siglec-4 in fact bound Neu5Acα2-3Galβ1-3(6S)GalNAc- and Neu5Acα2-6(Neu5Acα2-3Galβ1-3)GalNAc- stronger (Figure 4), suggesting the requirement of the negatively charged group on the core GalNAc. In addition, Siglec-4 from both human and rat bound the α2,8-sialic acid terminating glycan, which has not been observed previously.

Notably, none of the N-glycans on the CFG glycan microarray were among the highest binders. This may be due to multiple factors. First, most of the multi-sialylated highly branched N-glycans (i.e., tri- and tetraantennary) on the CFG array contain a bisecting GlcNAc (Table 1), a key structural feature that may alter glycan conformation and prevent glycan recognition. Second, the synthetic 2-amino-methyl N,O-hydroxyethyl linker which was used to immobilize all of these tri- and tetraantennary fully sialylated N-glycans may cause suboptimal presentation of the binding determinants, in comparison to the Asn-linked N-glycans on new N-glycan array.

#### Galectins

Galectins are another key family of GBPs implicated in virtually all aspects of the immune system (Brinchmann et al., 2018; Elola et al., 2018; Robinson et al., 2019). Galectins are expressed in many cell types including those of squamous epithelia, gastrointestinal tract, adipocytes, immune cells, and even erythrocytes (Thiemann and Baum, 2016). They share a common carbohydrate recognition domain (CRD) composed of two extended antiparallel β-sheets that fold into a β-sandwich structure forming the binding pocket. While certain amino acids within the CRDs are highly conserved (termed common carbohydrate-binding cassette) supporting the interaction with galactose, the variations outside of the binding cassette determine the fine specificities of different galectins. Knowledge of the fine binding preference of galectins can help identify the glycoproteins bearing these structures which in turn, facilitate the design of reagents that could specifically promote or prevent galectin binding activity.

We and others previously profiled in detail the binding specificities of many galectins to various Gal-containing glycans ranging from small monosaccharides to poly-LacNAc-containing glycoconjugates, with or without modifications of sialic acid, sulfation and blood group antigens (Hirabayashi et al., 2002; Leppänen et al., 2005; Stowell et al., 2008, 2010; Song et al., 2009; Horlacher et al., 2010). As evidence showed that some galectins can accommodate sialic acid (Leppänen et al., 2005; Stowell et al., 2008) and the N-glycan preferences of these galectins were not clear, we analyzed galectin-1 and -3 as examples on the newly generated N-glycan array.

Both of the galectins showed binding at 50 μg/mL (but not 5 μg/mL) to all of the LacNAc (Galβ1-4GlcNAc)-containing glycans, with or without core fucose (Figures 3B,D). Not surprisingly, the tetra-antennary N-glycans with four LacNAc sequences were most strongly bound. This observation, together with the absence of binding at lower protein concentration (Figure 3B), is reflective of the multivalent interactions of the two galectins with their glycan ligands. Our result clearly showed the tolerance of the two galectins to α2,3- but not to α2,6-linked sialic acid (Figure 3D), which has been reported in previous microarray studies using small glycan epitopes (Stowell et al., 2008) and cell-based assays (Patnaik et al., 2006; Stowell et al., 2008).

Moreover, our result identified the branching preference of galectin-1 and -3: the 2,2,4-form triantennary structures were always more strongly bound than the 2,2,6-isomeric triantennary counterparts; in galectin-3, the binding to 2,2,6-form triantennary structures was completely diminished (Figure 3D). This is a novel discovery in itself as MGAT5, the glycosyltransferase that initiates the β1,6-linked GlcNAc branch, was previously found to support the galectin-3 binding (Demetriou et al., 2001) on T-cells. Our results indicated that the expression of the 2,2,6-form triantennary N-glycan does not necessarily lead to the generation of the galectin-3 ligand. It is worth testing if the poly-LacNAc extension on either the 6- or the 4-linked arms affects galectin-3 binding.

It has been known that the human milk glycans (HMGs) are not significantly digested in the infant GI tract (Gnoth et al., 2000; Chaturvedi et al., 2001), besides lactose, which is typically digested prior to entering the small intestine. Thus, due to spatial correlation, HMGs can encounter and interact with galectins that are highly expressed locally. To explore these potential interactions, we took advantage of two glycan microarrays generated in house. One is a human milk shotgun glycan microarray containing 247 natural glycans purified from human milk, termed the HM-SGM array (Yu et al., 2014); the other is an array with defined, simple HMG structures, termed the HMG microarray. These arrays provide a unique collection of type 1 backbone (Galβ1-3GlcNAc)-containing glycans, which were underrepresented on other non-HMG glycan microarrays. All of the tested recombinant galectins, galectin-1,-3,-4,-7,-8, and -9, except for galectin-2, showed unique binding profiles on the HM-SGM array (Table 2). The majority of the binding signals were toward neutral glycan fractions or sialylated glycans with a non-sialylated branch (Table 2). These results were also corroborated by the HMG microarray and by isothermal titration microcalorimetry and hapten inhibition assays (Noll et al., 2016). This study extended these earlier observations and also identified more complex HMGs as additional targets of specific galectins.

**Table 2:** Major binding motifs bound by human galectins on the HMG array. Modified from Table II of Noll et al. (2016).

**Protein**	**HMG binding motif(s)**
Galectin-1	Galβ1-4GlcNAcβ1-6(Galβ1-3/4GlcNAcβ1-3)Galβ1-4Glc Galβ1-4GlcNAcβ1-6(Galβ1-3/4GlcNAcβ1-3)Galβ1-4GlcNAcβ1-
Galectin-3	Galβ1-4GlcNAcb1-3Galβ1-4GlcNAcb1-3Galβ1-4Glc
Galectin-4	Fucα1-2Galβ1-4GlcNAcb1-3Galβ1-4Glc Fucα1-2Galβ1-4GlcNAcb1-3Galβ1-4GlcNAcβ1-3
Galectin-7	Galβ1-3GlcNAcβ1-3Galβ1-4GlcNAcβ1-6(Galβ1-3GlcNAcβ1-3)Galβ1-4Glc
Galectin-8	Galβ1-4GlcNAcβ1-3Galβ1-4GlcNAcβ1-3Galβ1-4Glc
Galectin-9	Undefined neutral, nonfucosylated motif

*Motifs are proposed structures based on manual inspection of HMG-260 microarray data and the known HMG structure(s) within the bound samples*.

Collectively, these data highlighted the distinct binding specificities of galectins against the human milk metaglycome (Cummings and Pierce, 2014). This may at least partially account for the fact that each galectin has more or less unique physiological activities.

In summary, glycan microarray-based technology is not only useful for comparing binding patterns of different GBPs to deduce their specificities, it also supports the identification and characterization of novel carbohydrate ligands of the endogenous immune receptors. This information sheds light on the identification of the corresponding glycoprotein counterreceptors, and the functional intervention of these interactions.

### Discovery of Natural Glycan Ligands Involved in Infection and Microorganism Invasion

One major goal in the area of Functional Glycomics has been to identify naturally occurring host ligands for various microorganisms, which could help to understand their pathogenicity and infectivity. The CFG glycan array and other array platforms, such as the lipid-linked glycan array, have been used to study the glycan interactions with microorganisms such as bacteria and influenza viruses, as well as components of microorganisms that are important in various pathways of attachment and virulence, such as toxins, adhesins, and agglutinins (Childs et al., 2009; Liu et al., 2010; Petrova et al., 2016; Littler et al., 2017; Sun et al., 2018a; Yang et al., 2018). The glycan arrays, with both defined glycan structures as well as natural, undefined structures, have allowed for a deeper view of what glycan structures and classes play a role in the binding of relevant components or whole organisms.

As described above for galectins, the human milk glycan array has also been a critical tool for understanding microbial interactions, especially rotaviruses and influenza virus (Yu et al., 2012, 2014; Ashline et al., 2014; Hu et al., 2015; Sun et al., 2018a,b). We separated the soluble milk glycans into 247 different targets for printing on the array, which was one of the first instances of capturing all of the compounds of a “soluble glycome” for binding interaction purposes. Rotavirus VP domains were tested for their ability to bind, since rotaviruses are a main cause of gastrointestinal illness in infants and children. Interestingly, different VP proteins tested all bound to unique glycan structure subsets, many of which are believed to be non-metabolizable in the GI tract, leading to the hypothesis that these soluble glycans act as decoy receptors and prevent the rotavirus from attaching to the GI tract. These studies, in which the glycan arrays were a key tool, have been important to understanding the transmission and tropism of these viruses and the role that glycans play in the infection process.

Another example of the glycan array platform as a biochemical tool for ligand discovery has been the work with pig and human lung tissue for the main goal of searching for glycan ligands of influenza viruses. Sequence-defined glycan arrays have been utilized to assign the receptor-binding specificities of pandemic influenza viruses (H1N1) (Childs et al., 2009; Liu et al., 2010). Our initial studies focused on generating a “shotgun” or natural glycan microarray of the glycan materials isolated directly from pig lung tissue (Byrd-Leotis et al., 2014). This was the proof-of-concept that the tissue could be processed in such a way that the glycans could be isolated and printed for functional assays. Several 2,3- and 2,6-sialylated biantennary N-glycans were identified to be positively bound by influenza strains, which was an exciting development for studying endogenous glycan ligands. This led to the broad screening of various strains of influenza viruses, including these H3N2 strains that have progressively undergone “antigenic drift” in their head domains (Byrd-Leotis et al., 2019a) Surprisingly, the H3N2 drift strains almost completely lost their capacity in binding of canonical sialylated N-glycans on the sequence-defined N-glycan array (Byrd-Leotis et al., 2019a). Moreover, the discoveries with the pig lung glycan array opened the door to later studies with actual human lung tissue to search for endogenous receptors for influenza viruses (Byrd-Leotis et al., 2019b). Interestingly, it was discovered that many of the influenza viruses tested not only bound to sialylated structures, but also to phosphorylated glycans present in human lung tissue (Byrd-Leotis et al., 2019b). This novel finding will lead to brand new lines of study into viral pathogenesis and treatment options.

A complementary avenue to the “natural arrays” from human and other mammalian host tissues, is the generation of natural arrays from the microorganisms themselves. This strategy is important for being able to profile the innate and adaptive immune response toward the glycan-containing components of the organism. Our studies with *Schistosoma mansoni* and the human response to this parasitic infection led us to generate a natural shotgun N-glycan microarray from the schistosome egg glycoproteins (Mickum et al., 2016). The schistosome eggs, which were isolated from infected mice, were treated with PNGase F and A to isolate as many N-glycans as possible, separated and printed to create the arrays. These arrays, in conjunction with a defined schistosome-related glycan array (Luyai et al., 2014) were then interrogated with antibodies and sera from infected mammals. Both defined and shotgun arrays helped to define the FLDNF epitope (Fucα1-3GalNAcβ1-4(Fucα1-3)GlcNAc-), which was the target of a monoclonal antibody F2D2 derived from an infected mouse. Further, the arrays showed that the profile of the F2D2 antibody overlapped in distinct areas with infected rhesus monkeys and humans, leading to the presumption that the FLDNF glycan is a major antigenic target during schistosomiasis infections. The defined and shotgun array studies were complementary and informative, and helped to guide additional assays to characterize the immune response toward *S. mansoni* infection.

### Characterization of Natural Human Anti-glycome

The characterization of the human anti-glycome has benefited from both broad and specific glycan microarrays. The CFG microarray has been extensively utilized in studying the binding of GBPs including pathogenic toxins, intact viruses such as influenza and bacteria like *Escherichia coli*, various immune molecules including lectins and anti-carbohydrate antibodies (Blixt et al., 2004; Stowell et al., 2008; Hickey et al., 2010; Gulati et al., 2013; Jobling, 2016; Jones et al., 2016; Collins et al., 2017). As each array is conducted and data is collected, the results are published onto the CFG website, which is open to the public. This database is an invaluable resource for the glycobiology community, as it can inform researchers on which data has already been collected and point researchers in the right direction for additional experiments. Another useful resource produced by the CFG to screen mammalian GBPs is the Microbial Glycan Microarray (MGM). The most recent version of the array consists of 313 glycan targets from the polysaccharide material derived from different bacterial strains such as *E. coli, Proteus mirabilis, Pseudomonas aeruginosa, Providencia alcalifaciens, Providencia rustigianii, Providencia stuartii, Shigella boydii*, and *Shigella dysenteriae* (Bunker et al., 2017). These bacterial strains were largely provided by Dr. Yuri Knirel's group at the ND Zelinsky Institute of Organic Chemistry in Moscow, Russia. The bacterial polysaccharides presented on this array are coupled to NHS-activated glass microscope slides. Another microbe-focused glycan array has recently been developed by Seeberger group (Geissner et al., 2019). Complementary to the natural glycan approach, this new glycan array was generated by combining a variety of synthetic strategies. Together with the large collection of accumulated glycans from previous studies, the Max Planck Society (MPS) glycan library now contains more than 300 unique structures, making it one of the largest collections of microbial glycans. All of these microarray platforms have provided a unique resource to decipher the roles that certain proteins and antibodies (Bunker et al., 2017) may be playing in innate and adaptive immune detection of microbial carbohydrates.

Considering that the compounds for both of the CFG and MGM microarrays are limited in their quantity and accessibility, other defined arrays have been made using several classes of synthetic glycans such as α-glucans, β-glucans, xyloglucans, chitins, lacto-, globo-, and sialyl-series, gangliosides, blood groups A/B/O, Forssman and P antigen, Lewis antigen, Galα1-3R/Galili antigens, HNK1/GlcA, isoglobo, asialo-ganglio series, N-glycans and O-glycans. Many of these glycans have been shown to have biological relevance, and can be used for larger scale screenings. The NCFG has developed many of these arrays that are accessible to the public and is anticipated to serve as a key screening tool that is complementary to the CFG glycan array.

Each of these glycan microarrays has been used to characterize the specificity of human anti-glycan antibodies. Pooled IgG from healthy donors and IVIG (von Gunten et al., 2009; Schneider et al., 2015) have been profiled on a variety of different microarray platforms and have revealed a large diversity of anti-glycan antibody repertoires, including antibodies specific for cellulose and monosaccharides (Schwarz et al., 2003). Individual serum has been tested from healthy individuals (Muthana and Gildersleeve, 2016) as well as in HIV positive individuals (Scheepers et al., 2017) from the array platforms generated by Gildersleeve's group (see discussion in section Other Approaches in Glycan Microarray Generation). We have also surveyed the anti-carbohydrate antibody repertoire (ACAR) of 105 healthy donors, ranging from 20 to 60+ years old on the NCFGv1 array (Luetscher et al. in review). We discovered that the antibody profiles found within these individuals are relatively unique, with each individual displaying their own anti-carbohydrate signature. Additionally, we found that there are several glycan antigens that are highly immunogenic, with most individuals containing antibodies, which recognize the Forssman antigen, chitins and β-glucans. While it is difficult to compare certain aspects of the results of our study with Muthana and Gildersleeve (2016), as the glycan presentation and array platforms are not directly comparable, there were several common patterns of recognition by antibodies in both populations assayed. The NCFGv1 array has also been used to screen a cohort of Ugandan individuals with chronic exposure to *Mycobacterium tuberculosis* (Mtb), however these individuals never progress to active disease or result in a positive Mtb test. These resistant individuals were profiled for their anti-glycan antibody response, and compared to individuals with active and latent Mtb infections. The results of the NCFGv1 antibody profile from these distinct cohorts were indistinguishable, meaning that the resisters did not possess an anti-glycan antibody response that differed from active and latent patients. However, the data in its entirety has suggested that individuals resistant to Mtb infection have a unique and distinctive adaptive immune profile against Mtb specifically, although their deficiency in antibody response to carbohydrate antigens was eliminated by our results from the NCFGv1 array (Lu et al., 2019).

Understanding the repertoires of anti-glycan antibodies detected by glycan microarray analysis is important, and can be used to identify immunogens for glycoconjugate vaccines. This has been demonstrated by the development of glycan-based vaccines toward *Clostridium difficile* (Martin et al., 2013a,b), in which fragments of surface glycan PS-I were chemically synthesized and examined by microarray analyses with patient samples to identify the minimal antigenic epitopes. These candidates were coupled to carriers and yielded potent antigenic response in a mouse model.

### Plant Lectins and Antibodies as Essential Tools for Probing Sequence-Specific Glycans

Plant lectins and anti-glycan antibodies are essential tools in biological studies. The binding profiles of common plant lectins, which are naturally occurring GBPs, are very well-studied and they are vital for researchers identifying and characterizing specific glycan epitopes. Therefore, lectins are often used as sequence-specific controls in microarray assays.

Targeted glycan arrays have allowed for discovery of new information on the binding profiles of these already well-characterized lectins. For example, plant lectins have been utilized in detecting N-glycans based on what is currently known about their binding profiles. However, information on the exact relationship between the lectins and N-glycan variants is limited due to a lack of a variety of these structures on arrays. Using the new 32 N-Glycan Array created by Gao et al. (2019), each individual lectin was seen to have preferences for different N-glycan structures. Wheat germ agglutinin (WGA) and *Lens culinaris* agglutinin (LCA) bound the biantennary and 2,2,6- triantennary forms but not to the 2,2,4-counterparts or tetra-antennary N-glycans, which suggests that these lectins prefer branching; among the N-glycan backbone-binding lectins, only LCA, *Pisum sativum* agglutinin (PSA), and Concanavalin A (ConA) can accommodate both the α2,3- and α2,6-linked sialic acid. Other lectins, such as WGA, *Phaseolus vulgaris* erythroagglutinin (E-PHA), *Phaseolus vulgaris* leukoagglutinin (L-PHA), and *Datura stramonium* agglutinin (DSA) can only accommodate the α2,3-sialic acid (Gao et al., 2019). This level of detailed information was not previously known. Demonstrating these kinds of observable differences between binding to branched, core, and other modified glycans validates the necessity of expanding the repertoire of glycans available. With access to a number of slightly modified and varied structures, we can uncover new binding capabilities of lectins and expand upon the knowledge available about the lectin binding motifs.

As noted above, anti-glycan antibodies can be characterized on the array platforms, and many have been studied over the years on the CFG glycan array (Agrawal-Gamse et al., 2011; Zipser et al., 2012; Noble et al., 2013; Falkowska et al., 2014; Chua et al., 2015; Mickum et al., 2016; Tati et al., 2017; Nkurunungi et al., 2019), such as characterization of a new Lewis x antibody (Mandalasi et al., 2013) and multiple iterations of an anti-Tn antigen antibody (Chaturvedi et al., 2008; Tati et al., 2017). While antibody specificities are not the main focus of this review, it is clear from past work that the glycan microarrays have been and will continue to be very useful tools for their characterization, especially as the glycans and the types of linkers diversify.

### Glycan Array as a Platform for Developing Lamprey-Derived Smart Anti-glycan Reagents (SAGRs)

While glycan microarrays have been an essential tool for determining the specificity of a particular GBP, recently we have developed an alternative use of the technology to isolate and enrich for anti-glycan monoclonal antibodies. We have recently reported that the sea lamprey, *Petromyzon marinus*, generates a broad repertoire of anti-glycan antibodies in response to immunization with a variety of cell lines and biological tissues (McKitrick et al., in review). In contrast to the gnathostomes which produce antibodies made of immunoglobulin domains, the agnathans or jawless vertebrates, utilize a class of proteins constructed with leucine rich repeat motifs called Variable Lymphocyte Receptors (VLRs) as antigen receptors (Pancer et al., 2004; Alder et al., 2005, 2008; Boehm et al., 2018). These single-chain antibodies are produced and secreted by the VLRB lymphocyte lineage, and several groups have discovered and characterized monoclonal VLRB proteins that are specific for distinct carbohydrate structures (Han et al., 2008; Hong et al., 2013; Luo et al., 2013; Collins et al., 2017).

In order to utilize the VLRB proteins as traditional reagents in a laboratory setting, one must identify and enrich for VLRBs of a defined specificity and subsequently express them in a soluble form. To accomplish this, we as well as others have expressed the repertoire of VLRB proteins in a yeast surface display (YSD) platform (Xu et al., 2011; Velásquez et al., 2017; McKitrick et al., in review). We have then developed the methodology to incubate the VLRB expressing YSD library onto the glycan microarrays, where the VLRB antibodies will bind to the arrays in an antigen specific manner. The bound yeast colonies can then be transferred to solid media via replica plating, where monoclonals can be sequenced and screened individually for specificity (McKitrick et al., in review; McKitrick et al., accepted). Clones of interest are then expressed as a VLRB-Fc chimeric soluble protein, which we are calling Smart Anti-Glycan Reagents (SAGRs), and can be used for most routine research applications such as ELISA, western blotting etc. Thus, by combining YSD and glycan microarray technology, we have developed a high throughput methodology for the isolation and enrichment of glycan-specific reagents, which will greatly enhance the field of glycobiology.

## Other Approaches in Glycan Microarray Generation

### Diversity of Glycan Microarray Platforms

In addition to the widely used amine-based coupling method, other covalent attachment approaches have also been explored. One of the arrays that encompasses a large number of glycans is from the Gildersleeve group at NIH, in which glycans were covalently linked to a carrier protein bovine serum albumin (BSA) in the form of neoglycoproteins (Manimala et al., 2006). These glycoconjugates can be directly printed onto epoxy slides. The key feature of this array platform is multivalent presentation, which is similar to how natural glycans are found on glycoproteins. With a total of 60 amines, BSA has 54 that are expected to be solvent exposed (Oyelaran et al., 2009) and are fairly evenly distributed on the protein surface. The density of glycans on BSA can be controlled to some extent so that it becomes a multivalent scaffold to present glycans in a clustered or multivalent format to achieve high binding avidity. Thus, a neoglycoprotein-based glycan microarray has been developed and used in multiple studies, including the characterization of lectins (Luo et al., 2013) and monoclonal antibodies (Gildersleeve and Wright, 2016; Trabbic et al., 2019). In a recent screening of anti-glycan antibodies in the sera of pancreatic cancer patients, a glycan microarray containing 407 glyco-epitopes was generated and probed with sera from patients immunized with a whole cell vaccine. Antibody response was detected against many glycans including tumor-associated glycans, blood group antigen and α-Gal epitope (Xia et al., 2016). The anti-α-Gal antibodies, in particular, were inversely correlated with the overall survival rate and thus could be used as a potential biomarker to evaluate the immune responses to the vaccine.

In addition to the covalent methods, non-covalent attachment methods have also been developed and become the foundation of another comprehensive glycan microarray platform. Feizi and colleagues attached glycans to phospholipid carriers via reductive amination or oxime ligation (Tang et al., 1985; Chai et al., 2003; Liu et al., 2007; Li and Feizi, 2018). The products, neoglycolipids (NGLs) have hydrophobic lipid tails which facilitate immobilization on hydrophobic surfaces such as nitrocellulose through non-covalent hydrophobic interaction. This binding was so strong that the NGLs cannot be washed away by aqueous solution in microarray assays. Moreover, NGLs can be conveniently incorporated into liposomes supporting a clustered presentation. These NGL-carrying liposomes also enable microarray printing in aqueous solutions rather than in volatile solvents.

Since the generation of the first NGL-based glycan microarray (Fukui et al., 2002), the number and diversity of glycans in the libraries have been steadily growing (Palma et al., 2006; Gao et al., 2014; Li et al., 2017). Now there are more than 800 probes in their sequence-defined glycan microarray, making it one of the world largest resources (Li and Feizi, 2018). One of the recent applications of the NGL-based glycan microarray is the “Beam Search” which is an activity-chasing approach. In this effort, a minor O-glycan component from mucin-type glycoproteins was pinpointed and characterized as the binding ligand for rotaviruses (Li et al., 2017).

### New Concepts and Approaches in Glycan Microarray

#### Luminex/Multiplex Assays

As mentioned above, the glass slide-based glycan microarrays enable screening of GBPs against hundreds of structurally diverse glycans with minimal sample consumption. However, the traditional form of glycan microarrays on microscope glass slides is inconvenient in simultaneous analyses of multiple GBPs. Under different settings, one can probably investigate dozens (typically < 32) of samples in parallel. As a screening method, the glass slide-printed microarrays do not allow for analyses of large numbers (hundreds) of samples in a high-throughput manner. Recently, She et al. developed a multiplex glycan bead array platform based on the Luminex technology (Purohit et al., 2018). Each Luminex bead contains a unique fluorescent label among 500 labels that can be distinguished by the instrument. The binding assays can be easily performed in microwell plates, meaning the platform has the potential to simultaneous analyze 384 samples against up to 500 glycans in a single assay. The assembled bead array contained over 100 covalently attached glycans and was used in the studies of plant lectins, anti-glycan antibodies and human sera. Given the high-throughput and the prevalence of the Luminex instrument, this method could be adopted in clinical settings and benefit larger research and clinical community.

#### DNA-Coded Arrays

Several DNA-based glycan arrays have been generated (Chevolot et al., 2014; Yan et al., 2019). For example, Song et al. developed a DNA-coded glycan microarray platform by attaching glycans with DNA sequences and adopting next-generation sequencing (NGS) as the decoding method (Yan et al., 2019). Each glycan was tagged with a unique, defined oligonucleotide sequence. A library of DNA-coded glycans were mixed in a single vial, incubated with a biologically relevant GBP, then “pull down” bound glycans by microsphere beads immobilized with secondary antibody. For cells or intact microorganisms, bound glycans were “pulled down” by direct centrifugation. The DNA codes on bound glycans were sequenced by NGS, and the copy numbers of DNA codes represent relative binding intensities of the corresponding glycan structures. This microarray platform allows high throughput assays and the solution-phase binding assay is directly applicable to identifying glycan binding to intact cells (Yan et al., 2019).

#### Cell-Based Arrays

A cell-based platform has been reported in characterization of the structure of glycan ligands (Nonaka et al., 2014). A similar but more powerful and comprehensive approach was taken by Clausen and Yang's groups at the Copenhagen Center for Glycomics, where they developed a methodology to display the glycans on the surface of HEK293 or CHO cells. The overall approach has been to develop a panel of cell lines, genetically engineer to express a substantial portion of the human glycome (Narimatsu et al., 2019) and glycosaminoglycan or GAGome (Chen et al., 2018). Using a rather elegant combination of CRISPR/Cas9 knock-in and knock-outs of human glycosyltransferases (Narimatsu et al., 2018), the authors were able to drive the expression of unique glycan structures onto the cell surface glycoproteins. These panels of cell lines were then used to investigate the binding specificity of Siglecs, influenza viruses and Streptococcus adhesion proteins by flow cytometry. There are several advantages to using this approach, of particular note is the glycans on the cell surface are presented in the context of a fully folded protein, which is closest to their most native confirmation. In addition, upon identification of the binding profile of a given GBP, the full sequence of glycosyltransferases that are required to build the structure interacting with the GBP is also known, which provides a level of information that may not be given by the printed glycan microarrays. The authors have also developed the technology to secrete the glycans into the culture media, using the amino derivative XylNap,2-(6-((3-aminopropyl)oxy)-naphthyl)β-d-xylopyranoside (XylNapNH_2_) (Chen et al., 2018). Ultimately, the glycans are amine functionalized, and can be used for direct immobilization onto a microarray surface.

## Conclusions

As a robust high-throughput and high-content screening platform, glycan microarray represents a unique tool to define protein-glycan interactions in immunological studies. Glycan microarrays require minute amount of glycans that can be assayed with low background and high specificity. They enable functional screening and comparison in binding specificity of GBPs including antibodies, proteins, and viruses. Glycan microarrays provide information on both positively and negatively bound glycan structures which is particularly informative when placed in the relevant biological context.

Under current parameters, glycan microarray analyses cannot provide solutions to all scientific demands. For instance, this platform is not suitable for quantitative studies such as determining the binding constants. Several technical details are also yet to be addressed, and array robustness is related to the number and diversity of the printed compounds. The printing efficiency is generally low due to the efficiency in the underlying chemical reaction. The density and the presentation have also been shown to have a variable impact on the glycan interactions, and there may be cases where in-solution assays are more appropriate. Nonetheless, we and other groups have made many biologically important observations and advancements through glycan microarray interaction studies.

The glycan microarrays represent a *hypothesis-generating discovery tool* that can be effectively used to screen many types of immune proteins against hundreds of glycans simultaneously and provide leads to further experiments into these interactions. Sequence-defined and Shotgun microarrays are well complemented from distinct aspects of biological research: shotgun glycan arrays capture natural glycans that cannot be synthesized for defined arrays, sequence-defined glycan arrays corroborate the discovery made by shotgun glycan arrays. Therefore, they serve as a first-line screening that can guide further experimentation. An enormous amount of information can be gained from the array binding experiments themselves, especially if multiple types of arrays are utilized in a comparative fashion. They can also be coupled with creative steps such as enzyme treatments of the glycans, hapten inhibition, titration curves, and mutant proteins to obtain next-level binding characteristics. With increased sensitivity and throughput and the development of new formats including beads-based, cell-based and DNA-coded, glycan microarrays are now playing more and more important roles in discovering new GBPs and defining their essential functions in the host and microbiome interactome and in the immune system.

## Author Contributions

CG, MW, TM, AM, JH-M, and RC contributed to the writing and editing of the final review.

### Conflict of Interest

The authors declare that the research was conducted in the absence of any commercial or financial relationships that could be construed as a potential conflict of interest.
